# Comparison between autologous blood transfusion drainage and closed-suction drainage/no drainage in total knee arthroplasty: a meta-analysis

**DOI:** 10.1186/s12891-016-0993-z

**Published:** 2016-08-01

**Authors:** Kun-hao Hong, Jian-ke Pan, Wei-yi Yang, Ming-hui Luo, Shu-chai Xu, Jun Liu

**Affiliations:** 1 Department of Orthopedic Surgery, Guangdong Second Traditional Chinese Medicine Hospital, No. 60 Hengfu Road, Guangzhou, Guangdong 510095 China; 2 Department of Orthopedic Surgery, Second School of Clinical Medicine, Guangzhou University of Chinese Medicine, No. 111 Dade Road,, Guangzhou, Guangdong 510120 China

**Keywords:** Knee arthroplasty, Knee replacement, Autologous blood transfusion, Closed-suction, Drainage, Drains

## Abstract

**Background:**

Autologous blood transfusion (ABT) drainage system is a new unwashed salvaged blood retransfusion system for total knee replacement (TKA). However, whether to use ABT drainage, closed-suction (CS) drainage or no drainage in TKA surgery remains controversial. This is the first meta-analysis to assess the clinical efficiency, safety and potential advantages regarding the use of ABT drains compared with closed-suction/no drainage.

**Methods:**

PubMed, Embase, and the Cochrane Library were comprehensively searched in March 2015. Fifteen randomized controlled trials (RCTs) were identified and pooled for statistical analysis. The primary outcome evaluated was homologous blood transfusion rate. The secondary outcomes were post-operative haemoglobin on days 3–5, length of hospital stay and wound infections after TKA surgery.

**Results:**

The pooled data included 1,721 patients and showed that patients in the ABT drainage group might benefit from lower blood transfusion rates (16.59 % and 37.47 %, OR: 0.28 [0.14, 0.55]; 13.05 % and 16.91 %, OR: 0.73 [0.47,1.13], respectively). Autologous blood transfusion drainage and closed-suction drainage/no drainage have similar clinical efficacy and safety with regard to post-operative haemoglobin on days 3–5, length of hospital stay and wound infections.

**Conclusions:**

Autologous blood transfusion drainage offers a safe and efficient alternative to CS/no drainage with a lower blood transfusion rate. Future large-volume high-quality RCTs with extensive follow-up will affirm and update this system review.

**Electronic supplementary material:**

The online version of this article (doi:10.1186/s12891-016-0993-z) contains supplementary material, which is available to authorized users.

## Background

Total knee arthroplasty (TKA) is a highly successful standard procedure for patients who suffer serious knee arthralgia, instability and deformity. It is used after non-surgical treatments are exhausted, especially in advanced knee osteoarthritis [1, 2]. However, TKA can result in significant blood loss, reduction in haemoglobin (Hb) and other clinical risks [3, 4]. Reports of blood transfusion rates of 39 %–50 % have been published [5–7]. Autologous blood transfusion (ABT) drainage system is a new unwashed salvaged blood retransfusion system for primary TKA. However, whether to use ABT drainage, closed-suction (CS) drainage or no drainage in TKA surgery is still controversial. Some studies have found that ABT significantly reduced the need for homologous blood [8, 9], but other research has questioned the benefits of this method [10, 11] or demonstrated that post-TKA ABT had a limited effect on blood conservation [12, 13]. While gaining worldwide acceptance [14] for effectively decreasing hematoma formation [15, 16], conventional suction drains have been theoretically thought to decrease postoperative pain, swelling and incidence of infection [17]. However, a closed suction drainage system inevitably increases bleeding because the tamponade effect of a closed undrained wound is eliminated [14].

Until now, no systematic reviews incorporating meta-analyses (SRMA) have found sufficient evidence to recommend ABT drainage or no drainage in primary TKA. This is the first SRMA to systematically compare the clinical results of ABT drainage with closed-suction (CS)/no drainage in patients undergoing TKA. Previous SRMAs comparing ABT drainage versus CS drainage and CS drainage versus no drainage were published as the standard in evidence-based medicine with conflicting results [6, 18, 19]. Quinn et al. [19] showed that ABT drainage was superior to CS drainage for reducing blood transfusion rate (OR: 0.25 [0.13, 0.47]; *P* < 0.0001), and length of hospital stay (WMD: −0.25 [−0.48, −0.01]; *P* = 0.04). However, data extraction errors occurred in two included studies [20, 21] when extracting the number of patients requiring homologous blood transfusion for the meta-analysis. Another flaw is that in meta-analysis extracted data without intention-to-treat (ITT) analysis, treatment effectiveness may be exaggerated. The previous meta-analysis also did not evaluate other outcome measures like wound complication and post-operative haemoglobin on days 3–5. The aim of this SRMA was to pool extracted data from available published RCTs to provide a directly substantiated judgment regarding the use of ABT drainage following TKA surgery.

## Methods

In accordance with Preferred Reporting Items for Systematic Reviews and Meta-analysis (Additional file 1) [22], we made a prospective protocol of objectives, literature-search strategies, inclusion and exclusion criteria, outcome measurements and methods of statistical analysis before the research began.

### Data sources and search strategies

The following databases were searched in March 2015 without restriction to regions and publication types: Pubmed (1950–March 2015), Embase (1974–March 2015) and Cochrane Library (March 2015 Issue 3) (Additional file 2). The MeSH terms and their combinations searched in [Title/Abstract] was as follows: “total knee replacement” OR “total knee arthroplasty” OR “total knee prosthesis” OR “unicompartmental” OR “unicondylar” OR “arthroplasty, replacement, knee” [MeSH term] AND (“autologous blood transfusion” OR “autotransfusion” OR “blood transfusion, autologous” [MeSH Terms] OR “intraoperative blood salvage” OR “intraoperative blood” OR “postoperative blood salvage” OR “intraoperative blood cell salvage” OR “operative blood salvage” [MeSH Terms]). The reference lists of related reviews and original articles identified for any relevant studies, including randomized controlled trials (RCTs) involving adult humans were reviewed. The search also included the Controlled Trials Register (http://www.controlled-trials.com). Only articles originally written in English or translated into English were considered. When multiple reports describing the same situation were published, the most recent or complete report was used.

### Inclusion and exclusion criteria

Two independent researchers (Pan and Yang) identified studies that met the defined inclusion criteria, with disagreements resolved by consensus (Hong and Liu). Inclusion criteria were: (1) the comparison was between ABT drainage and CS/no drainage post TKA; (2) at least one of the quantitative outcomes we determined to evaluate was reported; (3) study design was a RCT; and (4) full text was published in English. Non-original research (e.g. review article, editorials, letter to the editor), case reports, animal experimental studies and duplicated publications were excluded.

### Data extraction and analysis

The data from eligible studies were extracted by two researchers (Hong and Pan) independently to minimize errors and reduce potential biases. In cases of disagreement, a consensus was reached by the adjudicating senior authors (Yang and Liu). The extracted data was input into a computerized spreadsheet, including sample size, study design, patient age, gender, preoperative/postoperative Hb levels, number of patients transfused with homologous blood, length of hospital stay and wound infection. The primary outcome was homologous blood transfusion rate. The secondary outcomes were post-operative haemoglobin on days 3–5, length of hospital stay and wound infection.

### Quality assessment and data synthesis

The RCTs were graded according to criteria of the Centre for Evidence-Based Medicine in Oxford, UK [23]. The quality of the RCTs; methodology was evaluated by the Cochrane risk of bias tool [24].

The statistical analysis was conducted with Cochrane Collaboration Review Manager 5.3.5 (Cochrane Collaboration, Oxford, UK). Our analyses were based on ITT or modified ITT data. Odds risk (OR) with 95 % confidence intervals (CIs) was calculated for dichotomous data and weighted mean differences (WMD) with 95 % CIs for continuous data. Statistical heterogeneity was assessed by using the chi-square test and I2 statistic. A random-effects model was used when significant heterogeneity was detected between studies without clinical diversity (*P* < 0.10; I2 > 50 %). Otherwise, a fixed-effect model was performed [24]. In cases with I^2^ values greater than 50 % for outcome measures, sensitivity analyses were conducted for heterogeneity. When overall results and conclusions are not affected by the different decisions that could be made during the review process, the results of the review can be regarded with a higher degree of certainty [24]. Funnel plots were used to identify potential publication bias.

## Results

**Fig. 1:**
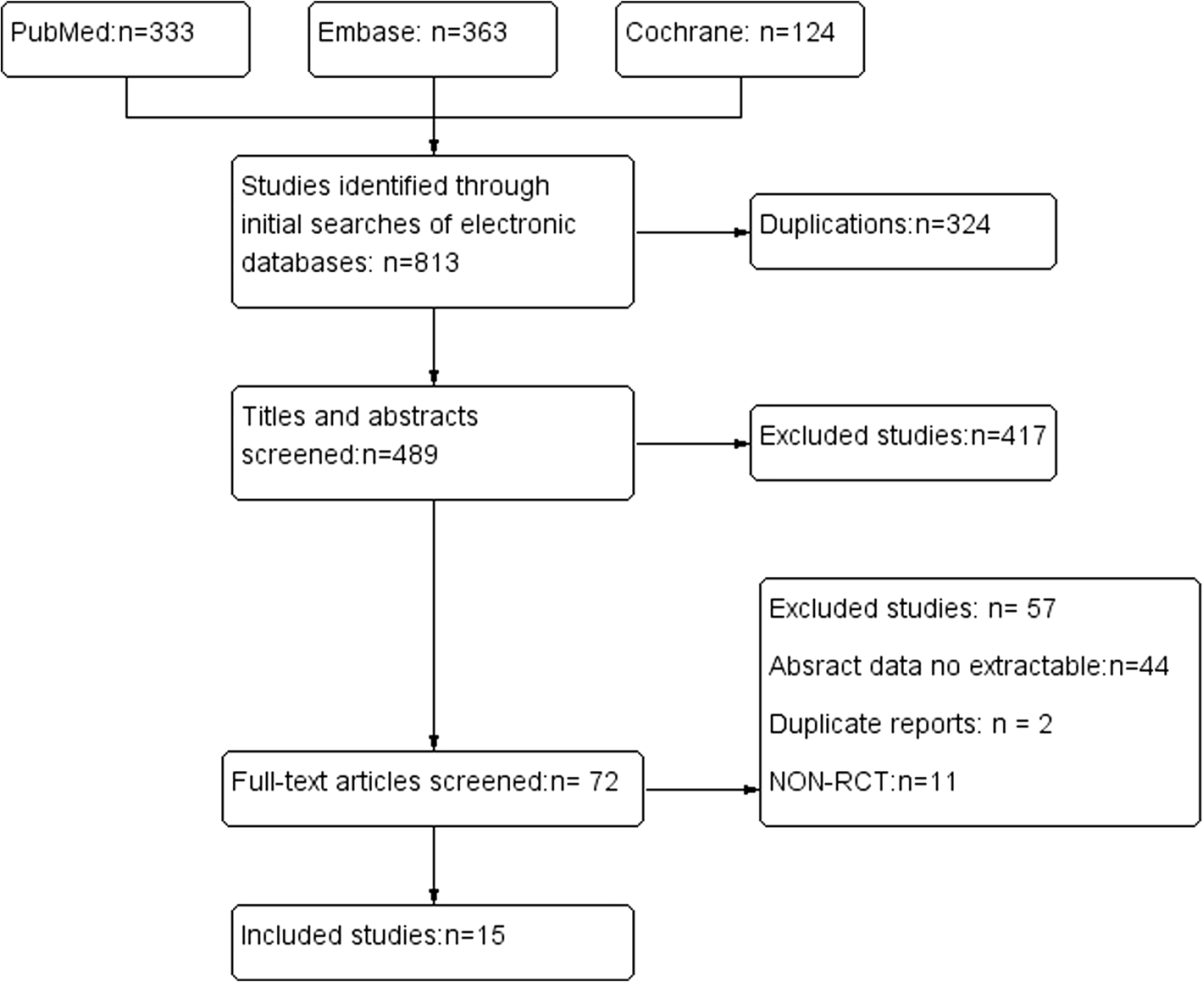
Flow diagram of studies identified, included and excluded ᅟ

Fifteen studies [10, 11, 20, 21, 25–35], all full-text articles in English including 1,721 cases (840 ABT drainage, 544 closed-suction drainage and 337 no drainage), were selected for synthesis analysis (Fig. 1, Table 1).

**Table 1:** Characteristics of included studies

Study	LOE^a^	Patients, no.			Surgical method	Age^a^			M:F ratio			Pre-op Hb^a^		
		A	B	C		A	B	C	A	B	C	A	B	C
Amin A 2008 [11]	1b	92	86	—	SU-TKA	70.3	70.4	—	43:49	39:47	—	13.2 (1.2)	13.4(1.3)	—
Zacharopoulos A 2007 [25]	2b	30	30	—	SU-TKA	69.2	70.2	—	6:24	7:23	—	NA	NA	—
Abuzakuk T 2007 [10]	1b	52	52	—	SU-TKA	NA	NA	—	21:31	22:30	—	13.6(1.5)	13.5(1.2)	—
Kirkos JM 2006 [27]	2b	78	77	—	SU-TKA	69.1(5.5)	68.9(5.1)	—	18:60	10:67	—	13.0(1.4)	13.1(1.4)	—
Dramis A 2006 [26]	2b	25	24	—	SU-TKA	NA	NA	—	NA	NA	—	NA	NA	—
Cheng SC 2005 [28]	1b	26	34	—	SU-TKA	72	69.6	—	6:20	12:22	—	12.4	12.8	—
Thomas D 2001 [29]	1b	115	116	—	SU-TKA	NA	NA	—	44:71	55:61	—	NA	NA	—
Adalberth G 1998 [20]	1b	30	30	30	SU-TKA	71(5.4)	72(8)	71(1.3)	NA	NA	NA	13.8(1.1)	14.3(1.3)	14.2(2.6)
Newman J 1997 [30]	2b	35	35	—	SU-TKA	NA	NA	—	NA	NA	—	13.4 ± 1.2	13.2 ± 1.4	—
Heddle NM 1992 [21]	1b	39	40	—	SU-TKA	69.3(6.9)	71(9)	—	25:14	26:14	—	NA	NA	—
Majkowski RS 1991 [31]	1b	20	20	—	SU-TKA	71.3	70.3	—	6:14	6:14	—	13.2	12.7	—
Horstmann W 2014 [33]	1b	59	—	56	SU-TKA	68(9)	—	69(8)	17:24		39:17	14(1.4)	—	14(1.4)
Dutton T 2012 [34]	2b	23	—	25	SU-TKA	68.7	—	70.5	10:13		10:15	NA	—	NA
Thomassen BJ 2014 [32]	1b	88	—	87	SU-TKA	68.9	—	69.5	NA	NA	NA	14.2	—	14.2
Ritter MA 1994 [35]	2b	128	—	123	SU-TKA	NA	—	NA	NA	NA	NA	13.0	—	13.1

*LOE* Level of evidence, *SU-TKA* selective unilateral total knee replacement, *B-TKA* bilateral total knee replacement

*A* autologous blood transfusion drainage, *B* conventional suction drain, *C* No drainage, *NA* data not available;. — = without this group ; ^a^Mean or Mean(SD)

### Characteristics of included studies

The demographic characteristics of the 15 studies are presented in Table 1.

**Fig. 2:**
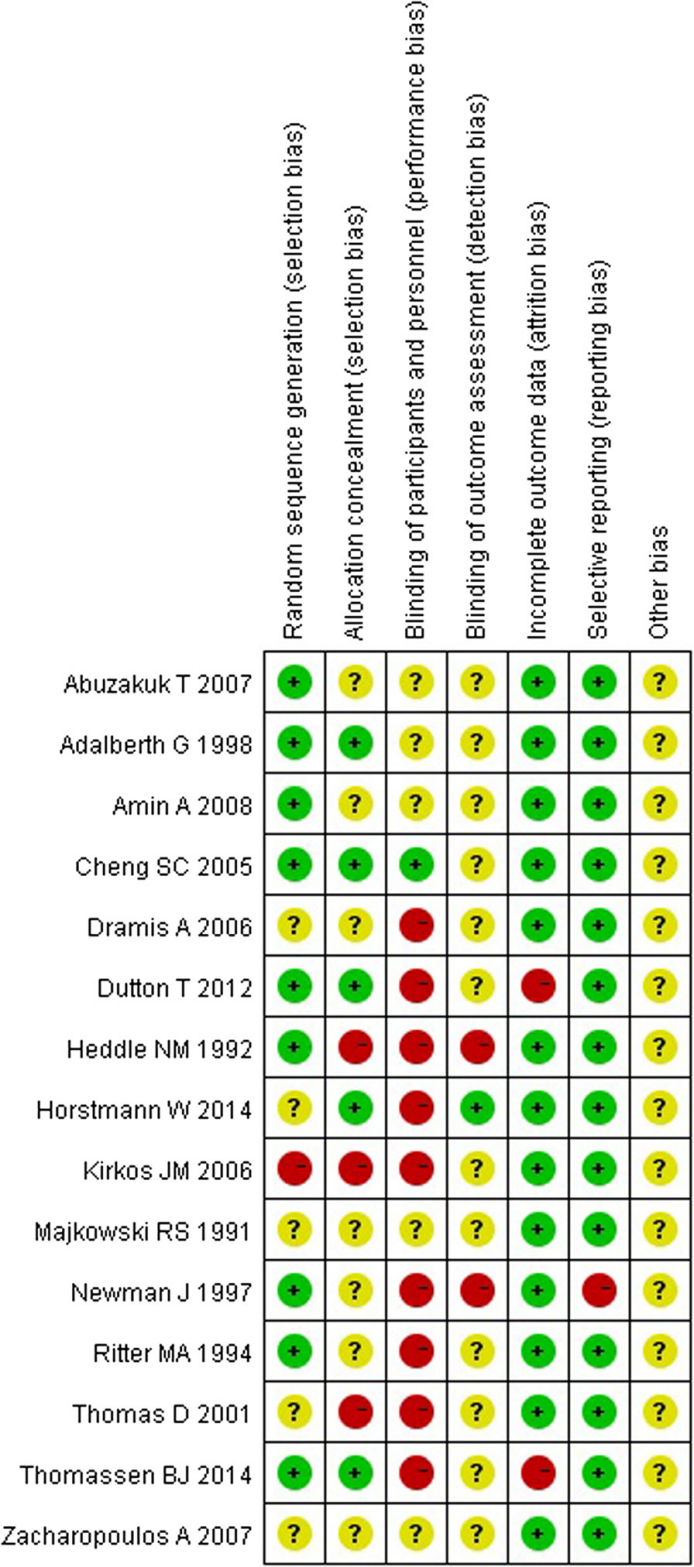
Risk of bias assessment

**Fig. 3:**
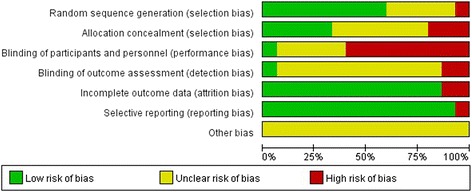
Risk of bias summary

The majority of the RCTs reviewed were moderate-quality studies. Among the included studies, there were nine RCTs [10, 11, 20, 21, 28, 29, 31–33] with a 1b level of evidence and six RCTs [25–27, 30, 34, 35] with a 2b level of evidence. Figures 2 and 3 showed the methodological quality of RCTs assessed by the Cochrane risk of bias tool. True randomization was used in only nine RCTs [10, 11, 20, 21, 28, 30, 32, 34, 35], while five RCTs [25, 26, 29, 31, 32] did not mention the method of randomization and one RCT [27] used quasi-randomization. Five studies [20, 28, 32–34] mentioned the method of allocation concealment. One study [28] provided information about blinding for participants. One study [33] mentioned the blinding of outcome assessments. Fourteen studies [10, 11, 20, 21, 25–29, 31–35] reported the complete analysis. One study [30] was at high risk on selective reporting.

### Primary outcomes

#### Homologous blood transfusion rate

**Fig. 4:**
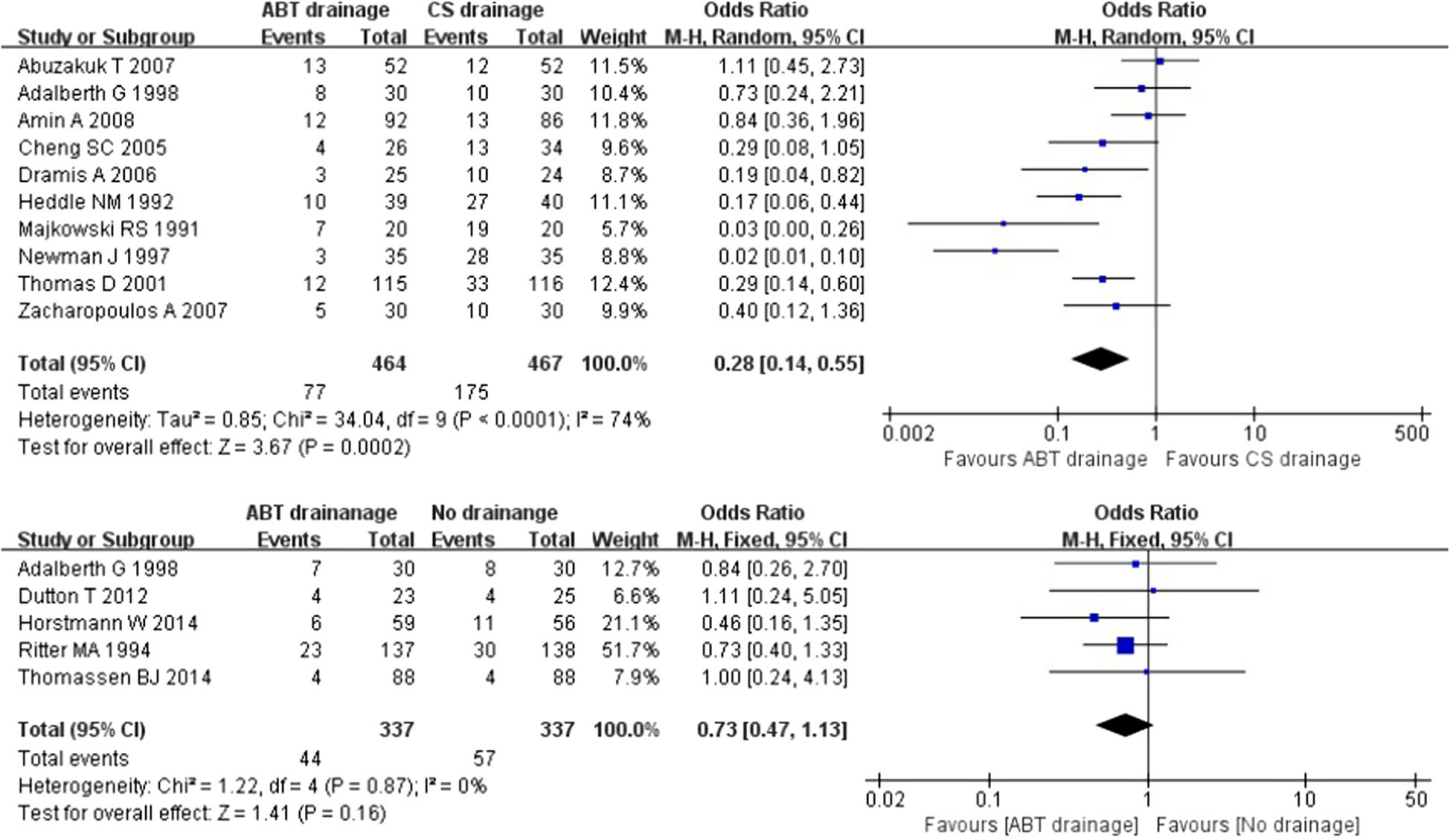
Forest plot and meta-analysis of homologous blood transfusion rate ᅟ

Fourteen studies [10, 11, 20, 21, 25, 26, 28–35] compared the effect of ABT drainage versus closed-suction drainage/no drainage according to changes in the number of patients requiring homologous blood transfusion. The meta–analysis of ABT versus CS drainage groups [10, 11, 20, 21, 25, 26, 28–31] showed substantial heterogeneity in the consistency of results (Chi^2^ = 34.04, *P* < 0.0001; I^2^ = 74 %). Sensitivity analyses were conducted with different decisions of excluding a study. When excluding the study [30] without clinical diversity detected, the heterogeneity was reduced (I^2^ = 59 %, *P* = 0.01). The result of sensitivity analysis was similar to the total analysis. Therefore, the random effects model showed a significant beneficial effect of ABT compared to CS drainage in reducing the blood transfusion rate (16.59 % and 37.47 %, OR: 0.28 [0.14, 0.55]; Z = 3.67, *P* < 0.0001) (Fig. 4). The meta-analysis of ABT versus no drainage groups [20, 32–35] showed no heterogeneity in the consistency of results (Chi^2^ = 1.22, *P* = 0.87; I^2^ = 0 %) and no significant difference in reducing the blood transfusion rate (13.05 % and 16.91 %, OR: 0.73 [0.47, 1.13], Z = 1.41, *P* = 0.16) (Fig. 4). However, a 3.86 % reduction in blood transfusion rate when comparing ABT drainage directly to no drainage should be given attention.

### Secondary outcomes

#### Post-operative haemoglobin on days 3–5

**Fig. 5:**
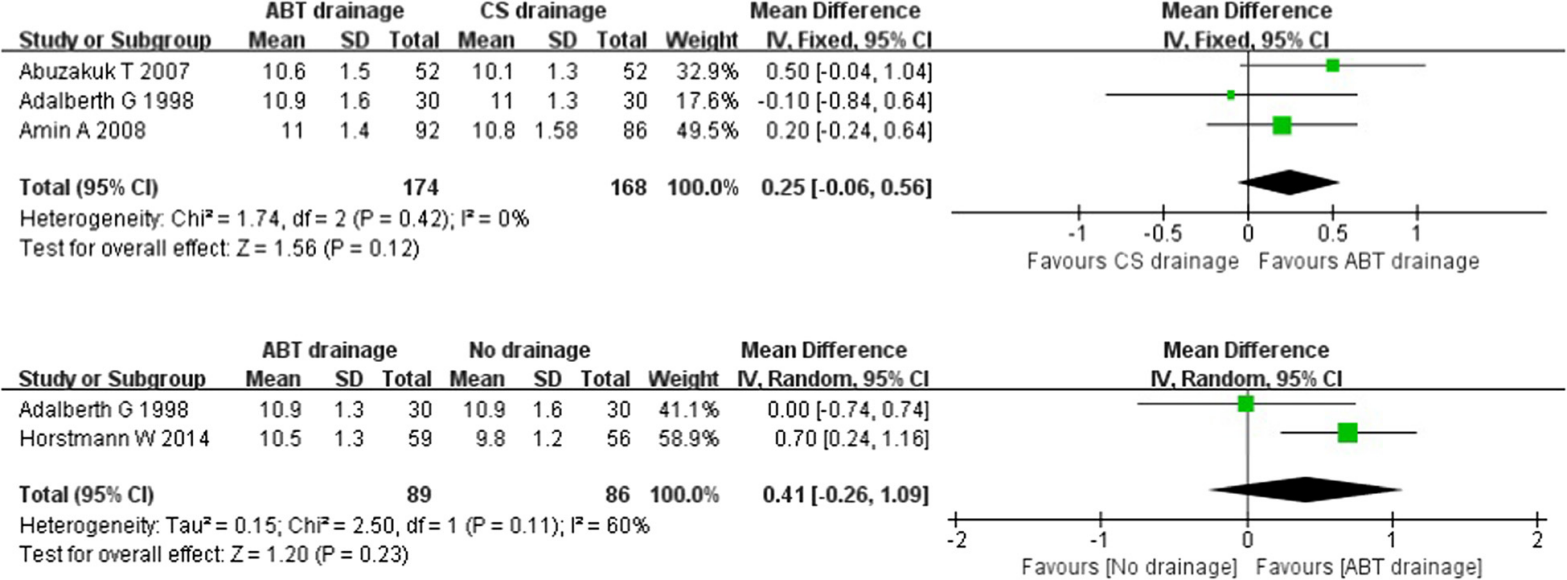
Forest plot and meta-analysis of post-operative haemoglobin days 3–5 ᅟ

Four studies [10, 11, 20, 33] reported post-operative haemoglobin on days 3–5. Among them, one study [10] only reported haemoglobin on the fifth day post-operation, while the other study reported haemoglobin only on the third day post-operation. Pooling the data of the 342 patients in the ABT versus CS drainage groups showed no significant difference (WMD: 0.25 [−0.06, 0.56] ; Z = 1.56, *P* = 1.2) (Fig. 5). No significant heterogeneity in this group was detected (*P* = 0.42, I^2^ = 0 %). The meta-analysis of ABT versus no drainage group showed substantial heterogeneity in the consistency of results (Chi^2^ = 2.50, *P* = 0.11; I^2^ = 60 %). For two studies, sensitivity analyses were not necessary with no clinical diversity identified. The random effects model of meta-analysis in the group showed no significant beneficial effect of ABT drainage compared with no drainage in post-operative haemoglobin on days 3–5 (WMD: 0.41 [−0.26, 1.09] ; Z = 1.20, *P* = 0.23).

### Length of hospital stay

**Fig. 6:**
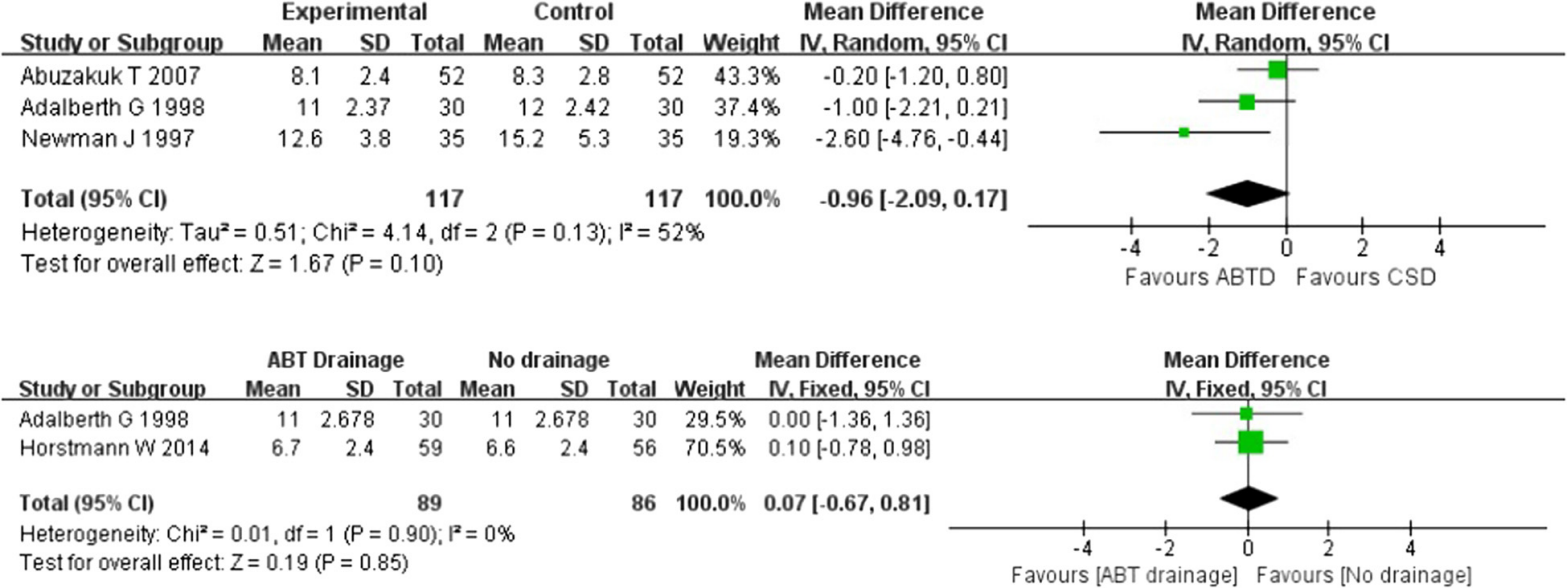
Forest plot and meta-analysis of length of hospital stay ᅟ

Pooling the data from four studies [10, 20, 30, 33] that assessed length of hospital stay in 339 patients showed no significant difference in the ABT versus CS drainage and ABT versus no drainage groups (WMD: −0.962 [−2.09, 0.17]; Z = 1.67, *P* = 0.01; WMD: 0.07 [−0.67, 0.81], Z = 0.19, *P* = 0.85, respectively). The comparison of ABT versus CS drainage group showed substantial heterogeneity in the consistency of trial results (Chi^2^ = 4.14, *P* = 0.13; I^2^ = 52 %). Owing to marked heterogeneity within the evaluated length of hospital stay, sensitivity analyses were conducted by excluding one study [10] with lower quality. Then, no significant heterogeneity was detected (*P* = 0.32, I^2^ = 0 %) and there was also no significant difference between the ABT and CS drainage groups in length of hospital stay (WMD: −0.52 [−1.30, 0.25]; Z = 1.33, *P* = 0.18). However, no significant heterogeneity was detected in the ABT drainage versus no drainage groups (Chi^2^ = 0.01, *P* = 0.90, I^2^ = 0 %). (Fig. 6).

### Wound infection

**Fig. 7:**
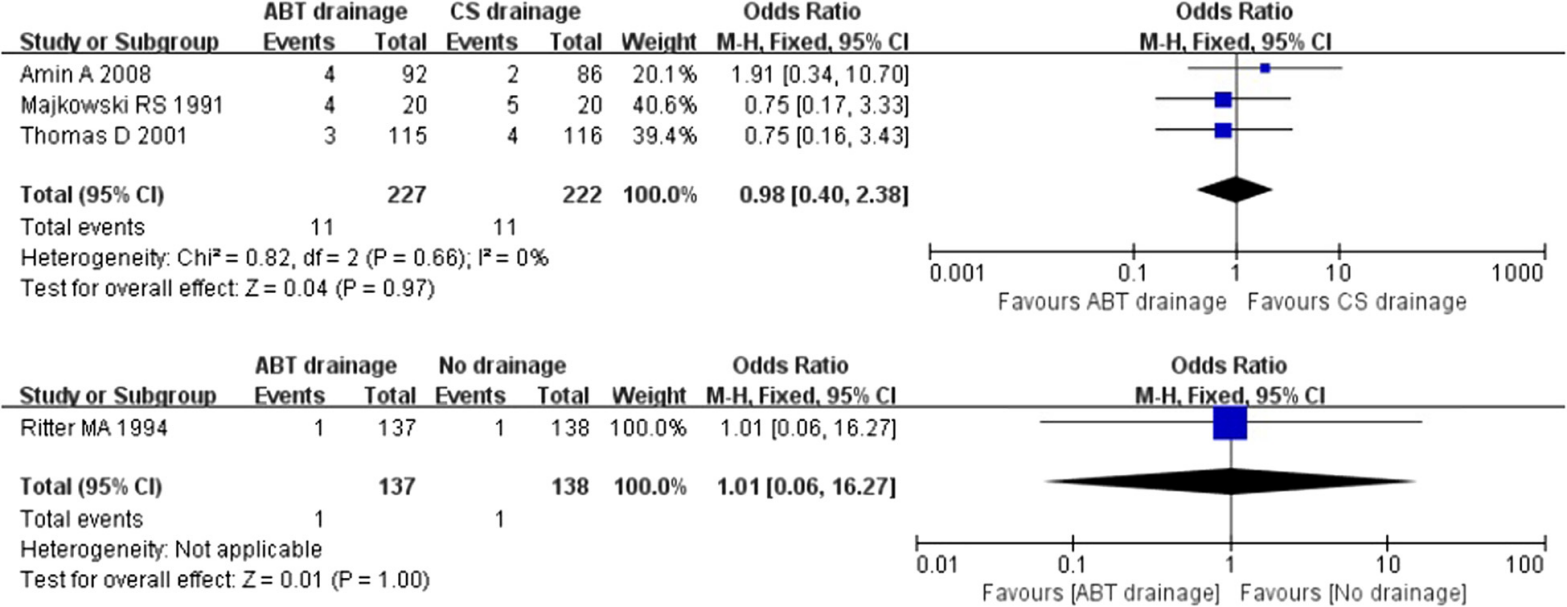
Forest plot and meta-analysis of wound infection ᅟ

Four studies [11, 29, 31, 35 ] reported the complication of wound infection. The result showed no heterogeneity in the consistency of results in ABT versus CS drainage groups (Chi^2^ = 0.80, *P* = 0.66; I^2^ = 0 %). Pooling the data of the 444 patients in the ABT versus CS drainage group and the 275 patients in the ABT versus no drainage group showed no significant difference between ABT drainage and closed-suction/no drainage (OR: −0.98 [0.40, 2.38] ; Z = 0.04, *P* = 0.97; OR: 1.01 [0.06, 16.27] , Z = 0.01, *P* = 1.00, respectively) (Fig. 7).

### Publication bias

**Fig. 8:**
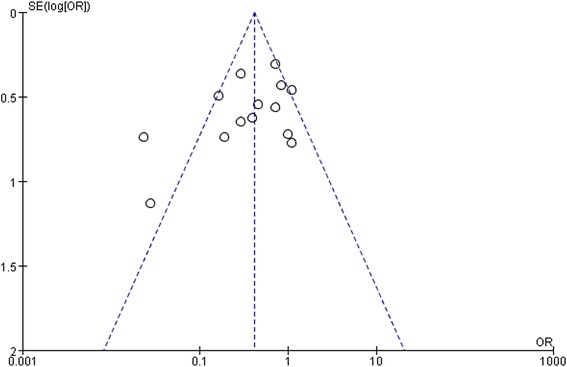
Funnel plot of homologous blood transfusion rate ᅟ

Figure 8 shows a funnel plot of the included studies that reported homologous blood transfusion rates. All studies lie inside the 95 % CIs except two studies, with an asymmetric distribution around the vertical indicating presence of obvious publication bias. This obvious publication bias is for the beneficial effect of lowering blood transfusion rate.

## Discussion

This SRMA of 15 studies including 1,721 patients comparing the clinical efficacy and safety of ABT drainage and closed-suction/no drainage showed significant statistical differences in homologous blood transfusion rates and similar clinical efficacy and safety in post-operative haemoglobin on days 3–5, length of hospital stay and wound infection in post-TKA patients.

With recent techniques, ABT drainage post TKA manifests the attractive concept of retransfusing collected drainage blood and continues to be a controversial issue in TKA surgery. Some studies have published considerable doubt with respect to its advantages [16, 36]. Despite the advantageous results, including reduced homologous blood transfusion rates shown in some studies [29, 37, 38], some authors have suggested insufficient efficiency for ABT [10, 39]. In spite of the paucity of consistent evidence, for many years the majority of orthopaedic procedures were followed by the use of ABT drainage post TKA to reduce the blood transfusion rate. However, the present systematic review and meta-analysis demonstrate a significant beneficial effect of ABT drainage in reducing the blood transfusion rate. The result of this meta-analysis showed no significant difference in post-operative haemoglobin on days 3–5. As those patients who received allogenic blood were not excluded from this analysis and there was a higher rate of allogenic blood transfusion in the closed suction drainage group compared with the ABT drainage group, it cannot be ascertained whether this is owing to a failure in ABT drainage to produce a beneficial effect on post-operative haemoglobin or to the positive nature of allogenic blood on haemoglobin levels. With the application of any new medical device, the safety of the patients is always of paramount importance. Acting as a channel for the introduction of infection, drainage may increase infection risk by impairing host resistance and allowing pathogens access to a sterile field [16, 17, 40]. The demands on nursing care and physiotherapy are increased to accommodate the presence of drainage. In orthopaedic surgery, wound infection is a devastating complication. However, the pooled data of postoperative outcomes indicated that the ABT drainage equipment was safe and effective for TKA. There was no significant difference in wound complication and length of hospital stay. This finding indicates that ABTD is as safe and efficient as CS/no drainage.

Some possible limitations of this meta-analysis and future research directions should be noted. The primary limitation is that the selected RCTs in this meta-analysis were moderate-quality studies with small sample sizes. With fewer included studies in the outcome analysis, the statistical heterogeneity assessments, including I^2^ text, were able to make false negative errors. Future systematic reviews should evaluate the indications from literature from sufficient, larger multi-centre clinical studies. In addition, this meta-analysis limited the included articles to those published in English. There might be selection bias in language. Finally, no long-term outcome measures were assessed, which is most pertinent to patients [41]. Therefore, other outcomes like range of movement, deep joint infection and component loosening, which are manifested after many years, should be considered.

## Conclusions

To our knowledge, this is the first SRMA to systematically compare the results of ABT drainage with closed-suction drainage/no drainage in patients undergoing TKA. The pooled results demonstrated that ABT drainage was more efficacious than CS drainage in clinically reducing blood transfusion rate. This meta-analysis also indicated that ABT drainage and closed-suction drainage/no drainage had similar clinical efficacy and safety with regard to post-operative haemoglobin on days 3–5, length of hospital stay and wound infection. Nevertheless, in spite of our rigorous methodology, the inherent limitations of eligible studies prevented us from reaching definitive conclusions. Based on the above clinical equipoise and potential benefit, future large-volume high-quality RCTs with long-term measures are awaited to affirm and update this system review.

## Additional files

Additional files 1: PRISMA checklist. (DOC 66 kb) Additional files 2: Search strategies. (DOC 78 kb)

## Abbreviations

TKA: total knee arthroplasty
ABT: autologous blood transfusion
CS: closed-suction
SRMA: systematic reviews incorporating meta-analyses
RCT: randomized controlled trial
ITT: intent-to-treat
OR: odds risk
WMD: weighted mean differences
CI: confidence interval
