# Supplementary material for: Comparison between autologous blood transfusion drainage and closed-suction drainage/no drainage in total knee arthroplasty: a meta-analysis
Source: BMC Musculoskelet Disord. 2016 Aug 1;17:142. doi: 10.1186/s12891-016-0993-z (PMC4968028; doi:10.1186/s12891-016-0993-z)
Supplement: Additional files 2: — Search strategies. (DOC 78 kb) [file 12891_2016_993_MOESM2_ESM.doc]

Searched in Pubmed

| Pubmed | 1950–March 2015 | **#1** | **(((((((((((((Operative Blood Salvage[MeSH Terms]) OR Blood Salvage, Operative[Title/Abstract]) OR Salvage, Operative Blood[Title/Abstract]) OR Intraoperative Blood Salvage[Title/Abstract]) OR Blood Salvage, Intraoperative[Title/Abstract]) OR Salvage, Intraoperative Blood[Title/Abstract]) OR Postoperative Blood Salvage[Title/Abstract]) OR Blood Salvage, Postoperative[Title/Abstract]) OR Salvage, Postoperative Blood[Title/Abstract]) OR Post-Operative Blood Salvage[Title/Abstract]) OR Blood Salvage, Post-Operative[Title/Abstract]) OR Post Operative Blood Salvage[Title/Abstract]) OR Salvage, Post-Operative Blood[Title/Abstract]) OR Intraoperative Blood Cell Salvage[Title/Abstract]** | **405** | |
| --- | --- | --- | --- | --- | --- |
|  |  | **#2** | Search **(((((((((Blood Transfusion, Autologous[MeSH Terms]) OR Transfusions, Autologous Blood[Title/Abstract]) OR Blood Transfusions, Autologous[Title/Abstract]) OR Autologous Blood Transfusions[Title/Abstract]) OR Autotransfusion[Title/Abstract]) OR Autotransfusions[Title/Abstract]) OR Transfusion, Autologous Blood[Title/Abstract]) OR Autologous Blood Transfusion[Title/Abstract]) OR ABT[Title/Abstract]) OR PAT[Title/Abstract]** | [**12555**](http://www.ncbi.nlm.nih.gov/pubmed/?cmd=HistorySearch&querykey=9) | |
|  |  | **#3** | **Search (((((((((((((((((((((((((((((((arthroplasty, replacement, knee[MeSH Terms]) OR Arthroplasties, Replacement, Knee[Title/Abstract]) OR Arthroplasty, Knee Replacement[Title/Abstract]) OR Knee Arthroplasty[Title/Abstract]) OR Arthroplasty, Knee[Title/Abstract]) OR Knee Replacement Arthroplasty[Title/Abstract]) OR Replacement Arthroplasties, Knee[Title/Abstract]) OR Replacement Arthroplasty, Knee[Title/Abstract]) OR Arthroplasties, Knee Replacement[Title/Abstract]) OR Knee Replacement Arthroplasties[Title/Abstract]) OR Arthroplasty, Replacement, Partial Knee[Title/Abstract]) OR Partial Knee Replacement[Title/Abstract]) OR Knee Replacement, Partial[Title/Abstract]) OR Unicondylar Knee Replacement[Title/Abstract]) OR Knee Replacement, Unicondylar[Title/Abstract]) OR Unicompartmental Knee Replacement[Title/Abstract]) OR Knee Replacement, Unicompartmental[Title/Abstract]) OR Unicondylar Knee Arthroplasty[Title/Abstract]) OR Arthroplasty, Unicondylar Knee[Title/Abstract]) OR Knee Arthroplasty, Unicondylar[Title/Abstract]) OR Partial Knee Arthroplasty[Title/Abstract]) OR Arthroplasty, Partial Knee[Title/Abstract]) OR Knee Arthroplasty, Partial[Title/Abstract]) OR Unicompartmental Knee Arthroplasty[Title/Abstract]) OR Arthroplasty, Unicompartmental Knee[Title/Abstract]) OR Knee Arthroplasty, Unicompartmental[Title/Abstract]) OR Knee Arthroplasty, Total[Title/Abstract]) OR Replacement, Total Knee[Title/Abstract]) OR Total Knee Replacement[Title/Abstract]) OR Knee Replacement, Total[Title/Abstract]) OR TKA[Title/Abstract]) OR TKR[Title/Abstract]** | **19930** | |
|  |  | **#4** | **#1 and #2 and #3** | | **333** |

Searched in Embase

| Embase | 1974–March 2015 | **#1** | **'blood autotransfusion'/exp OR 'blood autotransfusion' OR 'blood salvage'/exp OR 'blood salvage' OR 'blood transfusion, autologous'/exp OR 'blood transfusion, autologous' OR 'operative blood salvage'/exp OR 'operative blood salvage' OR 'transfusions, autologous blood' OR 'blood transfusions, autologous' OR 'autologous blood transfusions' OR 'autotransfusion'/exp OR 'autotransfusion' OR 'autotransfusions' OR 'transfusion, autologous blood' OR 'autologous blood transfusion'/exp OR 'autologous blood transfusion' OR 'blood salvage, operative' OR 'salvage, operative blood' OR 'intraoperative blood salvage' OR 'blood salvage, intraoperative' OR 'salvage, intraoperative blood' OR 'postoperative blood salvage' OR 'blood salvage, postoperative' OR 'salvage, postoperative blood' OR 'post-operative blood salvage' OR 'blood salvage, post-operative' OR 'post operative blood salvage' OR 'salvage, post-operative blood' OR 'intraoperative blood cell salvage' OR 'abt' OR 'pat' OR 'autotransfusion unit'/exp OR 'autotransfusion unit' AND [english]/lim** | 54793 |
| --- | --- | --- | --- | --- |
|  |  | **#2** | **knee arthroplasty'/exp OR 'knee arthroplasty' OR 'arthroplasty, replacement, knee'/exp OR 'arthroplasty, replacement, knee' OR 'arthroplasty, knee replacement' OR 'arthroplasty, knee'/exp OR 'arthroplasty, knee' OR 'knee replacement arthroplasty' OR 'replacement arthroplasties, knee' OR 'replacement arthroplasty, knee' OR 'arthroplasties, knee replacement' OR 'knee replacement arthroplasties' OR 'arthroplasty, replacement, partial knee' OR 'partial knee replacement' OR 'knee replacement, partial' OR 'unicondylar knee replacement' OR 'knee replacement, unicondylar' OR 'unicompartmental knee replacement' OR 'knee replacement, unicompartmental' OR 'unicondylar knee arthroplasty' OR 'arthroplasty, unicondylar knee' OR 'knee arthroplasty, unicondylar' OR 'partial knee arthroplasty' OR 'arthroplasty, partial knee' OR 'knee arthroplasty, partial' OR 'unicompartmental knee arthroplasty' OR 'arthroplasty, unicompartmental knee' OR 'knee arthroplasty, unicompartmental' OR 'knee arthroplasty, total'/exp OR 'knee arthroplasty, total' OR 'replacement, total knee' OR 'total knee replacement'/exp OR 'total knee replacement' OR 'knee replacement, total'/exp OR 'knee replacement, total' OR 'tka' OR 'tkr' AND [english]/lim** | 26231 |
|  |  | #3 | #1 AND #2 AND #3 | 363 |

Searched in Cochrane Library

| Cochrane Library | March 2015 Issue 3 | **#1** | **MeSH descriptor: [Blood Transfusion, Autologous] explode all trees** | **624** |
| --- | --- | --- | --- | --- |
|  |  | **#2** | **MeSH descriptor: [Operative Blood Salvage] explode all trees** | **18** |
|  |  | **#3** | **Blood Transfusion, Autologous or operative blood salvage or Transfusions, Autologous Blood or Blood Transfusions, Autologous or Autologous Blood Transfusions or Autotransfusion or Autotransfusions or Transfusion, Autologous Blood or Autologous Blood Transfusion or Blood Salvage, Operative or Salvage, Operative Blood or Intraoperative Blood Salvage or Blood Salvage, Intraoperative or Salvage, Intraoperative Blood or Postoperative Blood Salvage or Blood Salvage, Postoperative or Salvage, Postoperative Blood or Post-Operative Blood Salvage or Blood Salvage, Post-Operative or Post Operative Blood Salvage or Salvage, Post-Operative Blood or Intraoperative Blood Cell Salvage or ABT or PAT:ti,ab,kw (Word variations have been searched)** | **1575** |
|  |  | **#4** | **MeSH descriptor: [Arthroplasty, Replacement, Knee] explode all trees** | **1723** |
|  |  | **#5** | **Arthroplasties, Replacement, Knee or Arthroplasty, Knee Replacement or Knee Arthroplasty or Arthroplasty, Knee or Knee Replacement Arthroplasty or Replacement Arthroplasties, Knee or Replacement Arthroplasty, Knee or Arthroplasties, Knee Replacement or Knee Replacement Arthroplasties or Arthroplasty, Replacement, Partial Knee or Partial Knee Replacement or Knee Replacement, Partial or Unicondylar Knee Replacement or Knee Replacement, Unicondylar or Unicompartmental Knee Replacement or Knee Replacement, Unicompartmental or Unicondylar Knee Arthroplasty or Arthroplasty, Unicondylar Knee or Knee Arthroplasty, Unicondylar or Partial Knee Arthroplasty or Arthroplasty, Partial Knee or Knee Arthroplasty, Partial or Unicompartmental Knee Arthroplasty or Arthroplasty, Unicompartmental Knee or Knee Arthroplasty, Unicompartmental or Knee Arthroplasty, Total or Replacement, Total Knee or Total Knee Replacement or Knee Replacement, Total'or TKA or TKR:ti,ab,kw (Word variations have been searched)** | **3266** |
|  |  | **#6** | **#1 or #2 or #3** | **1575** |
|  |  | **#7** | **#4 or #5** | **3266** |
|  |  | **#8** | **#6 and #7** | **124** |
